# T cell repertoire profiling in allografts and native tissues in recipients with COVID–19 after solid organ transplantation: Insight into T cell–mediated allograft protection from viral infection

**DOI:** 10.3389/fimmu.2022.1056703

**Published:** 2022-12-14

**Authors:** Jianing Fu, Dylan Rust, Zhou Fang, Wenyu Jiao, Stephen Lagana, Ibrahim Batal, Bryan Chen, Sarah Merl, Rebecca Jones, Megan Sykes, Joshua Weiner

**Affiliations:** ^1^ Columbia Center for Translational Immunology, Department of Medicine, Columbia University, New York, NY, United States; ^2^ Columbia University Vagelos College of Physicians and Surgeons, New York, NY, United States; ^3^ Department of Pathology, Columbia University, New York, NY, United States; ^4^ Department of Microbiology and Immunology, Columbia University, New York, NY, United States; ^5^ Department of Surgery, Columbia University, New York, NY, United States

**Keywords:** COVID–19, T cell repertoire analysis, transplant recipients, immunosuppressed, viral response, immune response, T cell, SARS–CoV–2

## Abstract

**Introduction:**

The effects of the SARS-CoV-2 virus on the body, and why the effects are more severe in certain patients, remain incompletely understood. One population of special interest is transplant recipients because of their immunosuppressed state. Understanding the pathophysiology of graft dysfunction in transplant patients with the COVID-19 viral syndrome is important for prognosticating the risk to the graft as well as understanding how best to prevent and, if necessary, treat graft injury in these patients.

**Methods:**

We analyzed multiple types of solid organ transplant recipients (liver, kidney, heart or lung) at our institution who died from SARS-CoV-2 and underwent autopsy (n = 6) or whose grafts were biopsied during active SARS-CoV-2 infection (n = 8). Their serum inflammatory markers were examined together with the histological appearance, viral load, and TCR repertoire of their graft tissue and, for autopsy patients, several native tissues.

**Results:**

Histology and clinical lab results revealed a systemic inflammatory pattern that included elevated inflammatory markers and diffuse tissue damage regardless of graft rejection. Virus was detected throughout all tissues, although most abundant in lungs. The TCR repertoire was broadly similar throughout the tissues of each individual, with greater sharing of dominant clones associated with more rapid disease course. There was no difference in viral load or clonal distribution of overall, COVID-associated, or putative SARS-CoV-2-specific TCRs between allograft and native tissue. We further demonstrated that SARSCoV-2-specific TCR sequences in transplant patients lack a donor HLArestricted pattern, regardless of distribution in allograft or native tissues,suggesting that recognition of viral antigens on infiltrating recipient cells can effectively trigger host T cell anti-viral responses in both the host and graft.

**Discussion:**

Our findings suggest a systemic immune response to the SARS-CoV-2 virus in solid organ transplant patients that is not associated with rejection and consistent with a largely destructive effect of recipient HLA-restricted T cell clones that affects donor and native organs similarly.

## Introduction

Coronavirus disease 2019 (COVID-19), caused by severe acute respiratory syndrome coronavirus 2 (SARS-CoV-2), can produce a systemic inflammatory reaction involving extrapulmonary tissues and progress to multi-organ failure and death (1). The effects of the SARS-CoV-2 virus on the body, and why the effects are more severe in certain patients, remain incompletely understood. One population of special interest is transplant recipients because of their immunosuppressed state (2). Preliminary clinical data are mixed as to whether or not SARS-CoV2, like other viruses, causes more severe disease in transplant patients compared to nontransplant immunocompetent patients. Some indicate that transplant recipients have less severe disease (3), others show no differences in disease severity (4–6), while others report more severe morbidity (7) in transplant recipients. It is even conceivable that immunosuppressed transplant patients may be at less risk for severe manifestations resulting from cytokines and T cell responses (8), as calcineurin inhibitors and steroids, the mainstays of transplant immunosuppression, downregulate these pathways (9–11). Overall, the immune responses in SARS-CoV-2-infected transplant patients remain incompletely understood. It also remains unclear whether the allograft dysfunction that has been described in case reports of transplant recipients with the SARS-CoV-2 virus (12, 13) is mediated by viral damage, systemic inflammation, or an alloresponse (i.e., rejection). Understanding the pathophysiology of graft dysfunction in transplant patients with the COVID-19 viral syndrome is important for prognosticating the risk to the graft as well as understanding how best to prevent and, if necessary, treat graft injury in these patients.

The existing knowledge about the effects of the SARS-CoV-2 virus on transplanted organs is currently limited to histological findings in renal (14) and liver (15) allografts. Histological and virological investigations of other types of allograft, such as heart or lung, have not been reported, nor are there prior publications comparing the differential viral infiltration of grafts versus native organs. Adaptive T cell immune responses to the virus have not been compared in grafts and native tissue. T cell immunity plays a central role in the control of SARS-CoV-2 and determines clinical outcome (16). Given the potential for viral antigen presentation on donor vs recipient cells that may be extensively or even completely HLA-mismatched, the assessment of virus-specific T cell responses within a transplant recipient is particularly challenging. If the donor and recipient do not share HLA alleles, the ability of a presumably host HLA-restricted immune response to protect the graft from the virus is uncertain. While it has been shown that transplant patients are able to mount COVID-specific T cell responses following peptide stimulation (17, 18), there are no known studies of T cell clonal responses throughout the native and graft tissues in transplant patients using T cell receptor (TCR) sequencing. In fact, the collective published knowledge regarding viral loads throughout various tissues and T cell clonal responses is limited, even in the general non-immunosuppressed population, despite the comprehensive collection of the Human Cell Atlas initiative (www.humancellatlas.org).

We therefore aimed to characterize histology-proven tissue injury and viral infiltration of damaged allografts in different types of transplant recipients (liver, kidney, heart or lung) with severe COVID-19 infection, and we further integrated T cell clonal analysis in different types of allografts in comparison to a broad collection of native organs in autopsy specimens. We were able to perform this novel analysis because of our unique cohort of solid organ transplant patients infected by the SARS-CoV-2 virus, providing access to a range of native and graft samples from autopsy and biopsy. Beyond analysis of TCR clonal distribution in allograft versus native tissues, we were further able to identify COVID-associated TCRs in autopsy and biopsy by TCR beta chain CDR3 region DNA sequencing, in reference to the Adaptive Biotechnologies’ ImmunoSEQ^®^ COVID T-MAP™ database (19). Moreover, by applying the GLIPH2 algorithm (20) in reference to the immuneCODE™ MIRA database (MIRA: Multiplex Identification of Antigen-Specific TCR Assay) (21), we were additionally able to identify putative SARS-CoV-2-specific TCR sequences based on CDR3/V/J structural similarity at the amino acid level. Finally, we were able to gain deeper insights into HLA recognition and restriction of COVID-associated TCRs in allografts by referencing HLA typing information of subjects collected by the MIRA database, to investigate the levels of HLA sharing with our solid organ transplant patients and donors. Through these analyses, we achieve better understanding of the etiology of graft damage in transplant patients with COVID-19. In the process, we also derive lessons that are applicable to the general population and that shed light on our current understanding of the way the SARS-CoV-2 virus infects extrapulmonary tissues as well as the T cell responses to this systemic infection.

## Materials and methods

### Human subject recruitment and clinical protocols

Under an IRB-approved protocol (AAAT1929), we identified all solid organ transplants at our center between 2020-2021 who either died of SARS-CoV-2 infection and underwent autopsy or who received graft biopsy while infected with the SARS-CoV-2 virus. Clinical information (lab values, history, pathology reports, immunosuppressive regimen, etc.) was obtained from our electronic medical record system. All histology samples were obtained from tissue taken during autopsy or biopsy and stored in formalin-fixed paraffin-embedded (FFPE) blocks in our Pathology Department. Our Pathology collaborators confirmed the interpretation of the histology of each sample and provided digital images of the relevant samples.

### COVID PCR testing

COVID positivity within each tissue was determined by PCR performed by the Columbia University Microbiome Core. Since the weight of each sample was not known, allowing direct comparison of the copies per mass, the Core labeled each sample categorically as indeterminate, low positive, positive, and high positive.

### TCR-β CDR3 DNA sequencing and data analysis

FFPE specimens from Pts01-06 (autopsy) and Pts07-14 (biopsy) were provided by Department of Pathology, Columbia University and shipped at room temperature to Adaptive Biotechnologies for genomic DNA extraction and high-throughput TCR-β CDR3 sequencing. Sequence data were later retrieved from Adaptive Biotechnologies’ ImmunoSEQ software. PCR amplification, read sequencing, and mapping, with bias correction and internal controls, were performed by Adaptive Biotechnologies, returning tabulated template counts corresponding to unique productive (in frame) sequences across all samples. Unique sequence is defined by CDR3 + v region + j region at amino acid levels, which is referred to “bio_identity” by ImmunoSEQ software.

Sequencing data were analyzed using the integrated analysis toolset we developed for TCR repertoire analysis based on R language (22). Cumulative frequency was calculated as a percentage of all sequences weighted by copy numbers in designated populations (23). TCR repertoire diversity is assessed using clonality scores that are derived from Shannon’s entropy (24). Shannon’s entropy was calculated by summing the frequency of each productive sequence times the log (base 2) of the same frequency over all productive templates in a sample, which was normalized by the log (base 2) of the total number of unique productive sequences. This normalized entropy value is then inverted (1 - normalized entropy) to produce the clonality metric that ranges from 0 (the most diverse repertoire) to 1 (monoclonal distribution). R20 represents the minimum clone fraction required to capture 20% of overall clone frequency. R20 is obtained by first sorting clonal frequencies in decreasing order, then starting with the highest frequency clone and going in decreasing order to compute the fraction of all clones included in the top 20% of templates. R20 < 0.2 indicates non-uniform clone frequency, with extremely low values representing presence of a small number of highly dominant high-frequency clones. To measure of repertoire similarity which accounts for the frequencies of shared clones, Jensen-Shannon Divergence (JSD) was computed by summing of entropy(x) + entropy(y) divided by entropy of the summed vector x+y (25). JSD scales from 0 to 1 where 0 indicates identical repertoires with identical clone frequencies and 1 indicates no shared clones.

### Identification of COVID19-associated TCRs and putative SARS-CoV-2-specific TCRs

COVID19-associated TCRs were defined by sequence overlap (CDR3 + v + j) at amino acid levels of input TCRs from our transplant cohort with the ImmunoSEQ T-MAP COVID database (19). Putative SARS-CoV-2-specific TCRs were defined by applying the GLIPH2 (Grouping of Lymphocyte Interactions by Paratope Hotspots 2) clustering algorithm (20) in reference to the immuneCODE MIRA (multiplex identification of TCR antigen specificities) database (21). The GLIPH2 (20) algorithm was applied to each patient’s TCR dataset with the following parameters: local_min_pvalue=0.001, p_depth = 1000, global_convergence_cutoff = 1, simulation_depth=1000, kmer_min_depth=3, local_min_OVE=10, algorithm=GLIPH2, all_aa_interchangeable=1, refer_file=human_v2.0/ref_CD48_v2.0.txt, vb_score<0.05, length_score<0.05. HLA data were not included. This approach identified GLIPH2 clustering groups defined by local and global similarities. For a cluster based on local similarity (motif-based), motifs’ position within CDR3 are restricted within 3 amino acids. For a cluster based on global similarity, member CDR3s are of the same length and differ at the same position. The immuneCODE MIRA dataset (21) provide functional assay-validated SARS-CoV-2-specific TCRs and HLA typing information of relevant subjects. Publicly available ImmuneCODE-MIRA-Release002.1.zip file (peptide-detail-ci.csv, peptide-detail-cii.csv, minigene-detail.csv) was used to map SARS-CoV-2-specific TCRs to our dataset, and further identify “Putative SARS-CoV-2-specific TCRs” by integrating the GLIPH2 clustering data.

### HLA restriction analysis of SARS-CoV-2-specific TCRs identified by MIRA database

SARS-CoV-2-specific TCRs defined by sequence overlap with MIRA database were first grouped by their presence in both allograft and native tissues (“shared_Allo_Nat”), only in allograft (“Allo_only”) and only in native tissues (“Native only”) in autopsy specimens of Pts03, 04 and 06, from whom both donor (“D”) and recipient (“R”) HLA typing information is available. Given that all overlapping sequences from our cohort with MIRA database were with MHC class I restriction (**Supplemental Table 1**), we focused on HLA class I alleles (HLA-A, B, C) for restriction analysis. Overlapping HLA class I alleles between a patient (“D” and “R”) and MIRA subjects were annotated with donor HLA, recipient HLA, or donor/recipient shared HLA. Each sequence associated with at least one unique MIRA subject was defined by one of the putative categories: 1) “D restricted” if only annotated with donor HLA and the allele appears in all MIRA subjects with that unique TCR sequence; 2) “R restricted” if only annotated with recipient HLA and the allele appears in all MIRA subjects with that unique TCR sequence; 3) “R or D restricted” if annotated with both donor HLA and recipient HLA, with or without donor/recipient shared HLA alleles; 4) “unknow” restriction if with different HLA-A, B, C from both donor and recipient.

### Statistical analysis

GraphPad Prism was used to perform statistical analysis and determine statistical significance (p<0.05). One-way ANOVA, followed by Tukey’s multiple comparisons test was applied when statistical analysis involves one factor among three or more categorical groups, such as determining the differences of TCR repertoire similarity across tissue types of Pts01-06 measured by JSD. Statistical significance was determined by Two-way ANOVA, followed by Tukey’s multiple comparisons test when two factors are involved for multiple groups, such as organ types vs allograft or native tissue origins for Pts01-06, and organ types vs rejection or non-rejection for Pts07-14. Two-tailed paired Student’s t-test was applied when analyzing proportions of top 20 dominant TCR distribution in one designated tissue vs in all tissues within each subject of Pts01-06. Linear regression and R^2^ values were used to evaluate the association between two parameters.

## Results

### Severe SARS-CoV-2 infection in transplant recipients with graft dysfunction is characterized by diffuse tissue damage and elevated inflammatory markers

Our study includes a group of 6 transplant recipients (patient[Pt]01-Pt06) who succumbed to the SARS-CoV-2 infection and underwent autopsy and another group of 8 transplant recipients (Pt07-Pt14) with SARS-CoV-2 infection who were biopsied due to evidence of graft dysfunction. Their demographics and disease characteristics are summarized in **Tables 1** and **2**. For autopsy (Ax) patients, three were kidney recipients (KTx: Pts 03, 04, 06) and one each received lung (LuTx: Pt02), heart (HTx: Pt05), or liver (LiTx: Pt01) transplants. All except Pt04 were on at least 2 immunosuppressive agents (mycophenolate plus either tacrolimus, cyclosporine, or belatacept), and the lung recipient (Pt02) was additionally maintained on prednisone. Pt04 had 2 failed kidney grafts and had discontinued all immunosuppression prior to her SARS-CoV-2 infection due to frail health. For biopsy (Bx) patients, of whom six received KTx (Pts 07-12) and two received HTx (Pts 13-14), all were on at least 2 immunosuppressive agents.

**Table 1:** Patient Demographics.

	Age	Gender	Graft	Year of Transplant	Immunosuppressive Regimen
Patient 1	38	F	Liver	2017	Mycophenolate, Tacrolimus
Patient 2	65	M	Lung	2014	Mycophenolate, Cyclosporine, Prednisone
Patient 3	76	F	Kidney	2019	Mycophenolate, Tacrolimus
Patient 4	75	F	Kidney	1998, 2009	None
Patient 5	57	F	Heart	2000	Mycophenolate, Tacrolimus
Patient 6	71	M	Kidney	2017	Mycophenolate, Belatacept
Patient 7	56	M	Kidney	1999, 2003, 2020	Mycophenolate, Tacrolimus, Prednisone
Patient 8	52	M	Kidney	2019	Mycophenolate, Belatacept
Patient 9	72	M	Kidney	2020	Mycophenolate, Belatacept
Patient 10	55	F	Kidney	2020	Mycophenolate, Tacrolimus
Patient 11	53	F	Kidney	2019	Mycophenolate, Tacrolimus
Patient 12	66	M	Kidney	2020	Mycophenolate, Belatacept, Prednisone
Patient 13	53	M	Heart	2019	Mycophenolate, Tacrolimus, Prednisone
Patient 14	54	M	Heart	2018	Everolimus, Tacrolimus

For autopsy patients, who succumbed to their severe viral infection, inflammatory markers, where available, were almost universally markedly elevated, including IL-6, c-reactive protein (CRP), D-dimer, ferritin, lactate dehydrogenase (LDH), and white blood cell (WBC) count (**Table 2**), in line with previous reports (26). These markers were usually not checked in patients who received graft biopsy except in two patients (Pts 09 and 12) who had severe courses with prolonged intubation. The inflammatory markers were similarly elevated in those two patients.

**Table 2:** Patient Findings During SARS-CoV-2 Infection.

	Days from diagnosis to death	Transplanted Organ	Creatinine	AST	ALT	Alkaline Phosphatase	CK	Troponin	IL-6	CRP	ESR	Fibrinogen	D-dimer	Ferritin	LDH	WBC	Viral load	Pathology Findings	Rejection on Histology?
Units			mg/dL	units/L	units/L	units/L	units/L	ng/L	pg/mL	mg/L	mm/hour	mg/dL		ng/mL	units/L	x10^3/uL			
+Normal range			0.7-1.3	10-37	9-50	40-129	64-499	<=22	<=5	0-10	0-15	191-430	<=0.8 µg/mL	30-400	135-225	3.12-8.44			
Patient 1	22	Liver	1.85	196	103	206	162	112	>315	16.9	4	662	19	3777	1336	4.42	Heart-218 Lung-992,298 Kidney+Liver Graft-2500 Kidney-101	Heart-Interstitial edema, vacuolated myocytes, no inflammation Lung-Diffuse alveolar damage in RM lobe, organizing PNA RL lobe, mild congestion in upper lobes with edema and fibrin platelet aggregates, hemorrhae, emphysema Liver Graft-Minimal acute cellular rejection, mild inflammation with periportal fibrosis Kidney-Mild autolysis in cortex, tubular degenerative changes with brown casts, rare interstitial lymphocytes. No viral inclusions seen	Yes
Patient 2	22	Lung	1.82	421	546	220	612	74	>315	4.63	8	224	2.08	2853	1730	39.97	Heart-159 Lung Native-598,077 Lung Graft-1,342,438 Kidney+Liver-2345	Heart-Focal perivascular interstitial fibrosis Lung Native-Acute diffuse alveolar damage, microscopic honeycomb changes Lung Graft-Organizing diffuse alveolar damage, scattered interstitial inflammation with scattered T cells. Liver-Centrilobular congestion Kidney-Diffuse tubular injury, tubulointerstitial scarring, focal cortical infarct, fibrin thrombi	No
Patient 3	2	Kidney	1.2	88	90	82	N	N	N	N	N	N	N	N	N	13.87	Heart-0 Lung-140 Liver+Native Kidney-0 Kidney Graft-0	Heart-Myocyte hypertrophy with patchy interstitial fibrosis Lung-Congestion in all lobes with focal acute inflammation, organizing PNA in upper lobes Liver-Centrilobular congestion Kidney Native-Diffuse glomerularsclerosis and arteriosclerosis, thyroidization of tubules Kidney Graft-Mild arteriosclerosis	No
Patient 4	7	Kidney	0.87	26	10	115	N	560	N	166.5	N	N	N	5891	341	11.79	Heart-10 Lung-685,946 Liver-74 Kidney Native-42 Kidney Graft-24	Heart-Mild Chronic pericarditis Lung-Chronic and rare acute inflammation, scattered Sars-CoV-2 positive cells Liver-Significant sinusoidal dilation, vascular congestion and microvesicular steatosis Kidney Native-Near complete replacement of parenchyma with fluid-filled cysts Kidney Graft-Necrosis consistent with ischemic injury	No
Patient 5	16	Heart	2.47	11	7	86	72	63	589	77.3	26	395	1.95	704	584	17.82	Heart Graft-103 Lung-5511 Liver-88 Kidney-11	Heart Graft-Myocyte hypertrophy and degeneration, microthrombi, extensive epicardial fibrosis Lung-Acute alveolar damage with hyaline membrane and type II pneumocyte hyperplasia, microthrombi, organizing PNA Liver-Not commented on Kidney-Diabetic nephropathy with meangial sclerosis and severe arteriolar hyalinosis	No
Patient 6	33	Kidney	1.66	33	30	129	43	23	54	29.4	65	548	3.95	1054	588	21.64	Heart-8 Lung-88 Liver-22 Kidney Native-8 Kidney Graft-13	Heart-Diffuse fibrotic foci and myocyte hypertrophy Lung-Chronic and active alveloar damage with hyaline membrane, patchy infarct and microthrombi. Diffuse organizing PNA Liver-Foci of accumulated neutrophils in sinusoids. Background cystic changes Kidney Native-Diffuse cystic changes, periglomerular fibrosis, tubule thyroidization Kidney Graft-Mesangial expansion with FSGS, area of isometric tubular atrophy, mild cortical fibrosis, moderate vascular disease	No
Patient 7	N	Kidney	2.44	11	10	85	N	31	6.6	15.9	15	N	17.4	228	N	6.72	Kidney-25	Kidney Graft-Acute T cell mediated rejection, peritubular cappillaritis suggestive of early antibody mediated rejection, minimal. Immune complex mediated glomerulopathy	Yes
Patient 8	N	Kidney	1.68	14	11	91	N	N	N	N	N	N	N	N	N	6.36	Kidney-13	Kidney Graft-Severe intimal arteritis, severe interstitial inflammation and severe tubulitis consistent with acute T cell mediated rejection. Mild glomerulitis, mild tubular injury	Yes
Patient 9	N	Kidney	2.52	11	7	68	31	22	58.9	140.5	24	174	4.57	3394	214	10.92	Kidney-15	Kidney Graft-Acute tubular injury with vacuolization and many crystals. Focal endocapillary proliferative glomerularnephritis with masked IgG-Kappa deposits and focal glomerular fibrin	No
Patient 10	N	Kidney	2.45	15	11	138	N	N	N	N	N	N	N	N	N	3.47	Kidney-9	Kidney Graft-Tubular atrophy and interstitial fibrosis. Mild-moderate focal acute tubular injury and tubules with vacuolization. Interstitial tubulitis though 2/2 tacrolimus or Covid-19	No
Patient 11	N	Kidney	1.75	26	18	80	N	N	N	N	N	N	N	N	N	2.68	Kidney-13	Kidney Graft-Subacute thrombotic microangiopathy involving glomeruli	No
Patient 12	N	Kidney	1.33	11	5	226	10	43	32.6	25.3	85	400	1.47	1150	197	3.4	Kidney-10	Kidney Graft-Acute tubular injury with crystals. Focal mild-moderate peritubular capillaritis. Mild tubular atrophy and interstitial fibrosis.	No
Patient 13	N	Heart	1.28	31	33	115	N	N	N	N	N	N	N	N	N	7.7	Heart-27	Heart Graft-Perivascular lymphoid infiltrate	Yes
Patient 14	N	Heart	1.24	18	19	83	N	N	N	N	N	N	N	N	N	3.31	Heart-8	Heart Graft-No evidence of acute cellular rejection. No evidence of antibody mediated rejection	No

Red shading indicates values greatly over normal ranges; Light orange shading indicates values slightly over normal ranges.

Acute cellular rejection was identified on graft biopsy as the cause of renal dysfunction in 2 (Pts 07 and 08) of the 6 biopsy patients. Pt07 had inflammatory markers measured, which were moderately elevated, but was asymptomatic from the viral infection. The two biopsy patients (Pts 09 and 12) with severe courses and markedly increased inflammatory markers showed acute tubular injury on their graft biopsies. Two additional renal transplant recipients (Pts 10 and 11) received graft biopsy for elevated creatinine (Cr) but did not have evidence for severe COVID-19 or rejection. Their biopsies showed either calcineurin injury, which resolved when calcineurin inhibitor was switched to belatacept, or thrombotic microangiopathy. Thus, renal graft rejection was observed only in 2 patients (Pts 07 and 08) without severe COVID-19 disease, and remaining graft injury was due acute kidney injury in the setting of a significant inflammatory process.

**Figure 1:**
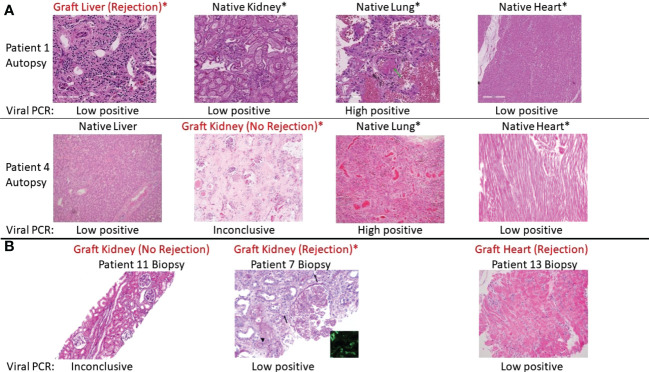
Representative histologic appearances of tissue from transplant patients with SARS-CoV-2 infection, including grafts with and without rejection. Viral PCR positivity is also shown below each picture. Asterix (*) indicates organ dysfunction as assessed by lab values or lack of function (e.g., requiring dialysis). **(A)** Various tissues (hematoxylin and eosin [H&E] stain) from autopsy of Patient 1, who had rejection of the liver graft during SARS-CoV-2 infection, and Patient 4, who did not have rejection of the kidney graft during SARS-CoV-2 infection. Patient 1 from left to right: graft liver with mild inflammation and periportal fibrosis consistent with acute cellular rejection. Native kidney with autolysis and tubular degenerative changes. Native lung with diffuse alveolar damage (green arrow), congestion, platelet aggregation, and hemorrhage. Native heart no inflammation. Patient 4 from left to right: native liver with centrilobular congestion. Graft kidney with ischemic changes and fibrosis but without significant inflammatory infiltrate. Native lung with congestion and focal inflammation. Native heart with mild chronic pericarditis (not captured in this image) but no interstitial inflammation. **(B)** Representative graft biopsies with and without rejection. From left to right: Patient 11 graft kidney showing tubules without significant inflammation (Jones methenamine silver). Patient 7 graft kidney with global endocapillary hypercellularity (transplant glomerulitis), peritubular capillaritis (arrows), and severe arteritis (arterial fibrin; arrowhead) (periodic acid–Schiff). These findings are accompanied by focal C4d staining in peritubular capillaries (inset, immunofluorescence staining for C4d, original magnification ×400). Patient 13 graft heart with perivascular lymphocytic infiltration consistent with rejection.

Among recipients of other transplanted organs who had COVID-19, the one liver graft in our cohort (Pt01) showed signs of mild acute cellular rejection. Native livers in two patients with severe COVID-19 showed evidence for injury, including transaminitis (Pts 02 and 03) and centrilobular congestion, likely related to hemodynamic instability and shock. Among heart grafts in patients with mild disease, a biopsy in one (Pt13) with new donor-specific antibody findings showed ISHLT 1R/1A rejection. The second was a routine biopsy in the setting of an asymptomatic SARS-CoV-2 infection and was unremarkable. All heart findings among the autopsy patients, including one transplanted heart (Pt05), showed chronic processes without acute findings. Finally, the lung graft in Pt02 showed severe inflammatory processes that mirrored those found in native lung. **Figure 1** shows representative histological findings for various organs in solid organ transplant recipients with SARS-CoV-2, including grafts with and without rejection.

In summary, our analyses in the context of COVID-19 infection reveal a systemic inflammatory pattern that included elevated inflammatory markers and diffuse tissue damage that was not usually associated with graft rejection based on histology in cases of severe infection.

### TCR clonal expansion and diversity in autopsy and biopsy specimens

We performed bulk TCR-β chain CDR3 sequencing on DNA from all native and allograft tissues from autopsy and biopsy specimens. Although the total number of productive templates as well as the number of unique sequences varied across individuals and tissue types (**Supplemental Figures 1A–D**), partially due to clinical sample availability, repertoire diversity and dominance measured by clonality and R20 were comparable between native tissue and allografts among autopsy specimens (**Supplemental Figures 1E, G**) and between rejecting and non-rejecting biopsies (**Supplemental Figures 1F, H**). Marked clonal expansion (clonality > 0.1 and R20 < 0.01) was observed in one kidney graft (Pt03) as well as one native kidney (Pt06) and one native lung (Pt03) specimen, with the remainder of tissues showing low level of clonal expansion.

### TCR sequences differ markedly between patients but are broadly similar between native organs and allograft within each patient

In the event of alloantigen-driven TCR clonal expansion and distribution, putative alloreactive TCRs are expected to be more abundant in the allograft than in native tissues, producing different distributions between host and donor tissues, as we have observed during intestinal allograft rejection (23, 27). On the other hand, SARS-CoV-2 infection may produce a systemic inflammatory reaction involving multiple organs and affecting graft and host tissues in the same way. Therefore, we next measured clonal overlap among the various tissue types to further distinguish between a systemic T cell response affecting all tissues versus an alloresponse restricted to the graft.

**Figure 2:**
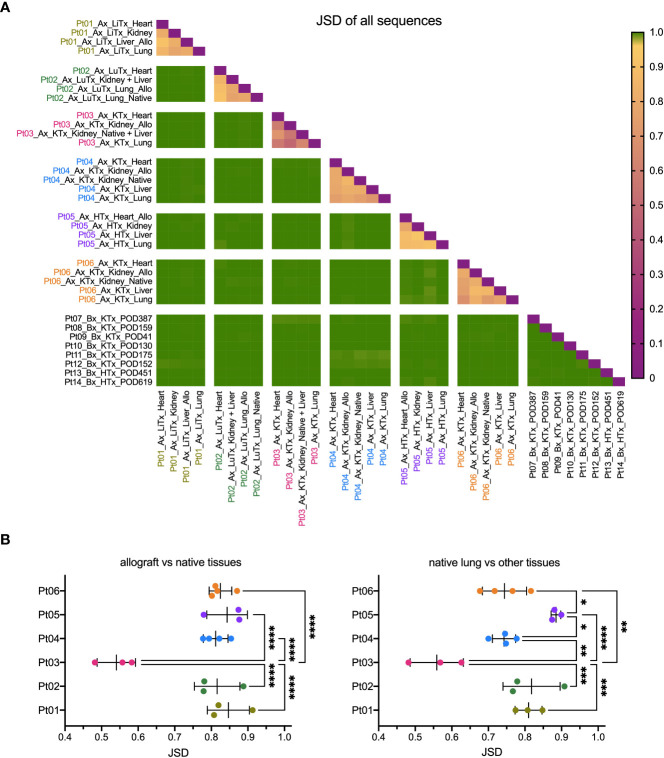
Similarity of TCR clonal distribution across individual patients and tissue types. **(A)** Jensen-Shannon divergence (JSD) quantitatively compares TCR repertoire overlap and similarity between two populations, taking into account clone frequency. JSD=1 indicates totally divergent repertoires and JSD=0 indicates identical repertoires. JSD data of all patients and tissue types were pooled in heatmap. Ax: autopsy. Bx: biopsy. LiTx: liver transplantation. LuTx: lung transplantation. KTx: kidney transplantation. HTx: heart transplantation. Allo: allograft. **(B)** JSD values between allograft vs native tissues (left panel) and between native lung vs other tissues (right panel) within each patient were pooled. One-way ANOVA, followed by Tukey’s multiple comparisons test was applied to determine statistical significance (*p<0.05, **p<0.01, ***p<0.001, ****p<0.0001).

We compared the similarity of TCR repertoires between patients and within the various tissues of each individual patient, including the allograft, by calculating the Jensen-Shannon divergence (JSD) index (**Figure 2**). JSD=1 indicates totally divergent repertoires and JSD=0 indicates identical repertoires (28, 29). As expected, based on the diverse HLA types, we identified largely distinct TCR repertoires between different patients. However, within each patient, the TCR repertoire sharing between tissues, both native and allograft, was significantly greater (**Figures 2A, B**). Specifically, JSD values are close to 1 (>0.95) when comparing any tissue from one patient to another. In comparison, significantly lower JSD values, ranging from 0.4 to 0.95, were identified when comparing the TCR repertoires between the native lung versus other native tissues or the allograft versus native tissues within each of the autopsy patients (**Figure 2B**). Interestingly, Pt03, who had the shortest clinical course between onset of COVID-19 symptoms and death from disease (**Table 2**), was an outlier with significantly lower JSD, indicating an even greater sharing of TCR clones across the body.

### Broad distribution of dominant clones correlates with rapid disease progression in transplant patients after COVID-19 diagnosis

**Figure 3:**
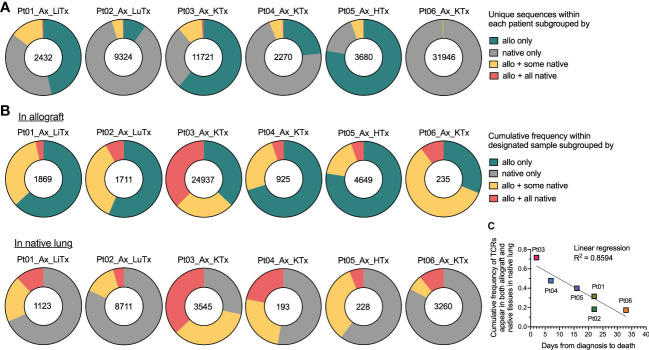
Broadness of TCR clonal distribution and its correlation with disease progression after COVID-19 diagnosis. **(A)** Quantification of the number of unique TCR sequences for each patient (shown in the center of each pie chart) present in the allograft (“allo”) only, native tissue (“native”) only, “allo + some native” tissues, and “allo + all native” tissues. **(B)** Quantification of the cumulative frequency of TCR sequences within the allograft (upper panel) or native lung (lower panel) of each patient subgrouped by their presence in “allo only”, “native only”, “allo + some native” and “allo + all native”. The number of total productive TCR templates in the allograft (upper panel) or native lung (lower panel) of individual patient is shown in the center of each pie chart. Cumulative frequency was calculated as a percentage of all sequences weighted by copy numbers in designated populations. **(C)** Association between cumulative frequency of TCR sequences appeared in both allograft and native tissues within native lung and days from COVID-19 diagnosis to death is shown. Linear regression R^2^ = 0.8594.

Since we observed a considerable level of repertoire similarity between various tissues within each patient, we next explored this sharing in greater detail by quantifying the number of clones present in 1) the allograft only, 2) native tissue only, 3) the allograft plus some native tissues, or 4) the allograft plus all native tissues (**Figure 3**). The fraction of unique TCR sequences shared between graft and native tissues was relatively low in all six autopsy patients (**Figure 3A**). However, when weighted by copy numbers of each unique sequence found in the allograft, there was 20-70% overlap between the graft and at least one of the native tissues (**Figure 3B**). Similarly, when considering the cumulative frequency of sequences found in the native lung, approximately 15-70% of sequences overlapped with the allograft and a significant fraction overlapped in all native tissues (**Figure 3B**). These data suggest that TCR sequences with high clonal dominance were widely distributed.

When we plotted the cumulative frequency of TCR sequences in the native lung that also appeared in both the allograft and native tissue versus the length of time between COVID-19 disease onset and death from disease, a linear inverse correlation with an R^2^ value of 0.8594 was detected (**Figure 3C**). The two patients (Pts 03 and 04) with the highest percentage of clones in the native lung that were shared with the allograft and native tissues were notable for having the shortest diagnosis to death times among our autopsy cohort (2 and 7 days respectively versus 16-33 days for the other patients). As with the JSD result, this observation suggests that a systemic T cell response may be associated with faster disease course.

### Distribution of top dominant clones in autopsy patients is more suggestive of a systemic response than an alloresponse

**Figure 4:**
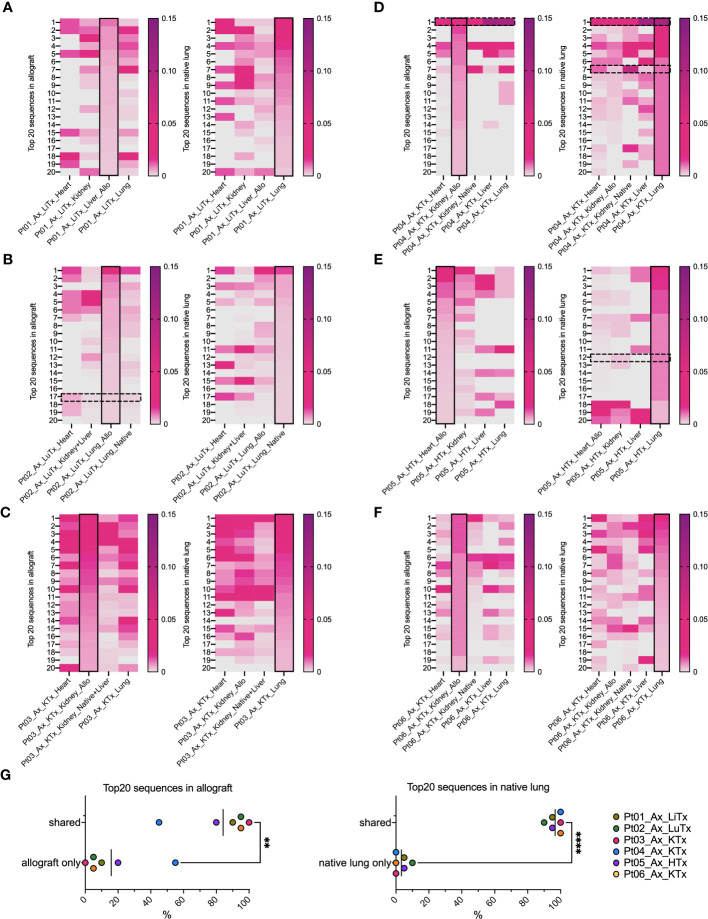
Top 20 dominant TCR clonal distribution and overlap in allografts and native lungs in individual patient’s autopsy specimens. In each heatmap panel **(A–F**: Pts01-06), solid black rectangle in column highlights top 20 dominant TCRs in each row ranking by frequency (ranges from 0-0.15) within that designated sample, either allograft (left panel) or native lung (right panel) of each patient. Black dashed line rectangles indicate rows with COVID-associated TCR sequences detectable in Pts02, 04, 05 in reference to ImmunoSEQ T-MAP COVID database. **(G)** Percentages of top 20 dominant clones in allograft present in “allograft only” and “shared” by allograft and native tissues were summarized in left panel. Percentages of top 20 dominant clones in native lung present in “native lung only” and “shared” by allograft and native tissues were summarized in right panel. Paired t test was applied to determine statistical significance (**p<0.01, ****p<0.0001).

When we focused on the top 20 most frequent clones in each tissue (**Figure 4**), the majority in native lung were found to be shared across a variety of tissue types in each patient, regardless of allogeneic vs native origin (**Figures 4A–G**). Similarly, the majority of the top 20 clones found in the allograft were also found in native tissues (**Figures 4A–F**, left panel, **Figure 4G**). This was even the case for Pt01, who was the only patient in our autopsy cohort to have histological evidence of rejection, suggesting that the immune response within the graft may have been part of, or triggered by, a systemic process.

### Distribution of COVID-associated TCRs and their association with SARS-CoV-2 viral loads across individuals and tissue types are indicative of a systemic response

**Figure 5:**
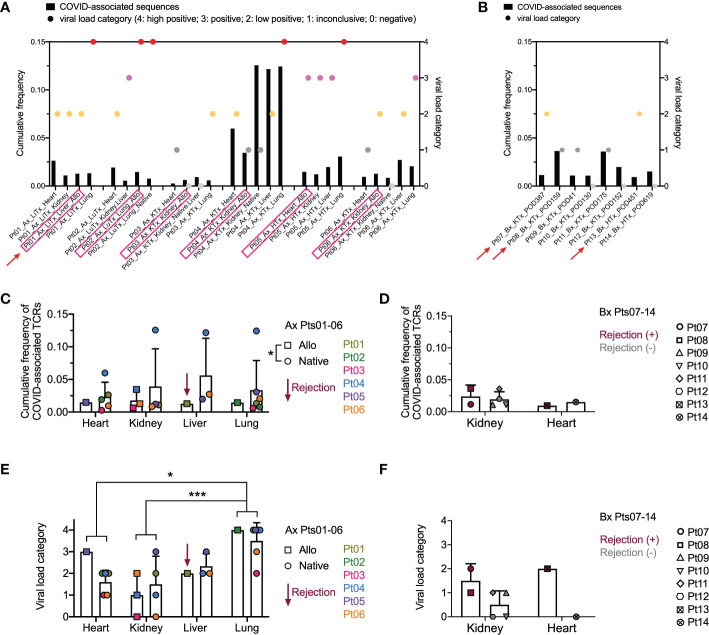
Overview of COVID-associated TCR clonal distribution and association with viral loads across individual patients and tissue types. **(A, B)** Left Y axis: cumulative frequency of COVID-associated TCRs defined by CDR3 + v + j sequence overlap at the amino acid level with the ImmunoSEQ T-MAP COVID database. Right Y axis: semi-quantified viral load categories (4: high positive; 3: positive; 2: low positive; 1: inconclusive; 0: negative). Autopsy (Ax) and biopsy (Bx) specimens are shown in A and B, respectively, with allograft samples highlighted with pink rectangles on the x axis. Red arrows indicate graft rejection determined by histology. **(C)** Cumulative frequency of COVID-associated TCRs in different organs subgrouped by allografts (squares) and native tissues (circles) for autopsy specimens of Pts01-06. Pt01 liver graft was diagnosed as rejection. **(D)** Cumulative frequency of COVID-associated TCRs in kidney or heart biopsies of Pts07-14 subgrouped by rejection status. **(E)** Viral load categories of different organs subgrouped by allografts (squares) and native tissues (circles) for autopsy specimens of Pts01-06. **(F)** Viral load categories of kidney or heart biopsies of Pts07-14 subgrouped by rejection status. Statistical significance was determined by Two-way ANOVA, followed by Tukey’s multiple comparisons test for panel C-F. *p<0.05, ***p<0.001.

Since the data in **Figures 3**, **4** suggested that a systemic process affected the allograft, native tissues, and lungs, we addressed the possibility that dominant T cell clones in these tissues were SARS-CoV-2 antigen-specific. To identify COVID-associated TCRs, we cross-referenced the TCR sequences in our analysis with the ImmunoSEQ^®^ COVID T-MAP™ database (19) through bioidentity (CDR3+V+J amino acid) overlap. Despite constituting a small subset of all productive templates in each sample (**Supplemental Figures 2A–D** compared to **Supplemental Figures 1A–D**), COVID-associated TCRs were identifiable in each tissue in all patients we interrogated, including autopsies (**Supplemental Figures 2A, C**) and biopsies (**Supplemental Figures 2B, D**). A few of the top 20 dominant sequences were identified as COVID-associated (**Figures 4B–E**) in 3 out of 6 autopsy patients, including the top dominant clone within the native lung of Pt04. We then associated the cumulative frequency of COVID-associated TCRs with the viral load within each tissue as assessed by PCR (**Figures 5A, B**). SARS-CoV-2 viral load was semi-quantified into five categories with a score from 4 to 0, respectively: high positive, positive, low positive, inconclusive and negative. As has been established with non-transplant patients with the SARS-CoV-2 virus (30), we found that the lungs were the most markedly virally-positive tissue in our cohort of transplant patients with severe SARS-CoV-2 infection, regardless of whether the lungs were native or allogeneic (**Figures 5A, E**). Virus was also found in almost all extra-pulmonary tissues (**Table 2** and **Figure 5A**), with no significant difference in cumulative frequency of COVID-associated sequences or viral load between allografts and native organs (with or without rejection) or between different types of tissues other than significantly higher viral load in lung compared to kidney and heart (**Figures 5C–F**). Similar to the distribution of total TCR clones (**Figure 3**), COVID-associated TCR clones were widely shared between native and allograft tissues, especially when measured by cumulative frequency (**Supplemental Figures 3A, B**).

### Putative SARS-CoV-2-specific TCRs identified by GLIPH2 and their association with viral loads across individuals and tissue types

**Figure 6:**
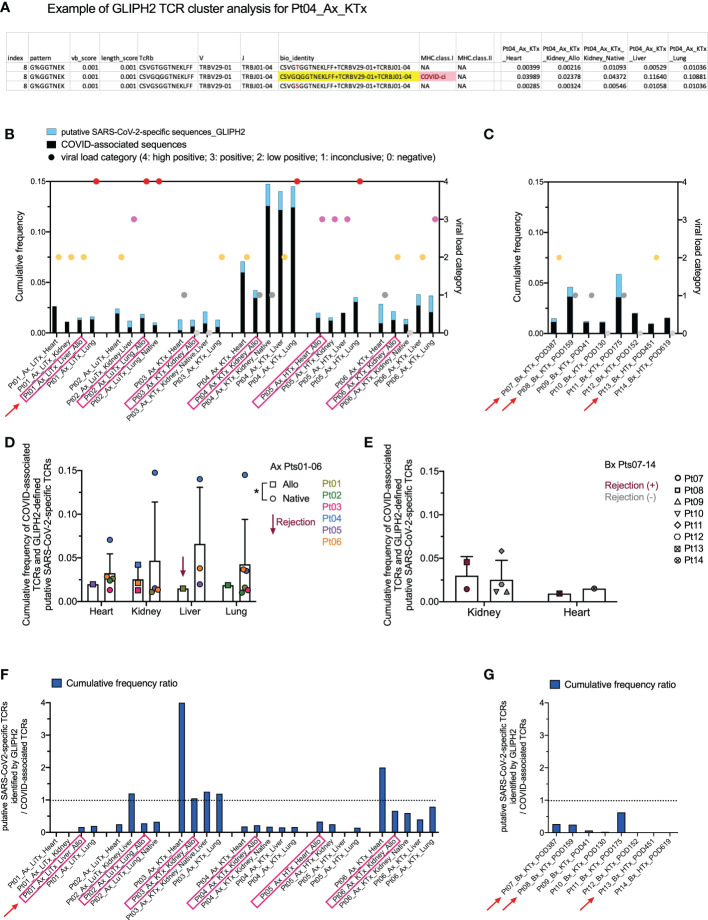
Identification of putative SARS-CoV-2-specific TCRs and their clonal distribution across individual patients and tissue types. **(A)** A representative example of defining putative SARS-CoV-2-specific TCRs in Pt04 by applying the GLIPH2 clustering algorithm in reference to the immuneCODE™ MIRA database is shown in the table. Three TCRs, each with a unique CDR3 + v + j sequence at the amino acid level (bio_identity), formed a cluster (Index 8) by sharing the same structural pattern of “G%GGTNEK”. One TCR highlighted in yellow in the bio_identity column overlaps with the sequence validated as SARS-CoV-2-specific TCR with MHC class I restriction in the MIRA database. The other two TCRs in this cluster, which are termed as “putative SARS-CoV-2-specific TCRs”, differ with one amino acid highlighted in red compared to the reference TCR. Cumulative frequencies of each TCR in different autopsy specimens of Pt04 are shown in the last five columns of the table. **(B, C)** Left Y axis: combined cumulative frequency of COVID-associated TCRs (black bars) and GLIPH2-defined putative SARS-CoV-2-specific TCRs (light blue bars). Right Y axis: viral load categories. Autopsy and biopsy specimens are shown in B and C, respectively, with allograft samples highlighted with pink rectangles and red arrows indicate graft rejection determined by histology. **(D)** Cumulative frequency of COVID-associated TCRs and GLIPH2-defined putative SARS-CoV-2-specific TCRs in different organs subgrouped by allografts (squares) and native tissues (circles) for autopsy specimens of Pts01-06. **(E)** Cumulative frequency of COVID-associated TCRs and GLIPH2-defined putative SARS-CoV-2-specific TCRs in kidney or heart biopsies of Pts07-14 subgrouped by rejection status. No statistical significance was determined by Two-way ANOVA, followed by Tukey’s multiple comparisons test for panel D-E. Cumulative frequency ratios of GLIPH2-defined putative SARS-CoV-2-specific TCRs vs COVID-associated TCRs in autopsy **(F)** and biopsy **(G)** specimens are shown.

While the COVID T-MAP™ database provides useful information, our data also indicate that its utility is restricted by containing only a limited number of donors with limited HLA alleles. For example, Pt03, the patient with the shortest diagnosis to death course, appeared to have the greatest sharing of the top 20 dominant pulmonary sequences throughout extrapulmonary tissues, but none of these sequences were identified as COVID-associated by mapping to the COVID T-MAP™ database (**Figure 4C**), and only up to 1% of TCRs in allograft and native tissues of this patient were identified as COVID-associated (**Figure 5A**). Because the COVID T-MAP™ database is limited to TCRs that have been validated thus far to be SARS-CoV-2-reactive, we sought to expand the definition of such clones by performing TCR clustering analysis using the GLIPH2 algorithm in reference to the immuneCODE™ MIRA database (21). We thereby sought to add TCR sequences that might additionally be COVID-associated based on their structural similarity with pre-defined SARS-CoV-2-specific TCRs by MIRA assay, which we termed “putative SARS-CoV-2-specific TCRs.” The MIRA database provides a collection of validated SARS-CoV-2-specific TCRs with MHC class I or class II restriction. In a representative TCR cluster identified by GLIPH2 in Pt04 (**Figure 6A**), one TCR overlap with the MIRA database formed a cluster with another two TCRs, each with the same V and J family but a single amino acid difference in the CDR3 region with the reference TCR, and showed a broad tissue distribution in both the allograft and all native tissues tested.

We next added the cumulative frequency of all putative SARS-CoV-2-specific TCR sequences identified by GLIPH2 to the validated ImmunoSEQ^®^ COVID T-MAP™ database of COVID-associated sequences (**Figures 6B, C**). The combined cumulative frequency was comparable regardless of tissue type, allograft or native origin, or presence of rejection (**Figures 6D, E**). As with the COVID-associated TCR sequences identified by clonal overlap with the COVID T-MAP™ database, the unique putative SARS-CoV-2-specific TCR sequences added by GLIPH2 analysis were shared between native and allograft organs within most autopsy patients except Pt01 (**Supplemental Figure 4**). The cumulative frequency ratio of the additional sequences to the original sequences was calculated. GLIPH2 analysis added an especially large proportion (>50%) of additional sequences in Pt03 (**Figures 6F, G**). Pts 02, 06, and 11 also had relatively large proportions of sequences added (**Figures 6F, G**). The addition of GLIPH2 analysis produced an especially large increase in cumulative frequency of COVID-associated sequences in the heart in some samples (Pts 03 and 06), making the value comparable with other tissue sites, including the lung (**Figure 6F**). This was especially striking given the low overall TCR repertoire and low viral level in heart samples. For three of the six autopsy patients (Pt01-03), the heart specimens had by far the lowest number of total TCR templates and unique sequences detected, and the viral PCR (**Table 2**; **Figure 6B**) was no more than inconclusive or low positive in any heart specimen except one (Pt05) (**Figure 5A**). Despite this, the cumulative frequency of COVID-associated clones in the heart was higher than any other sampled tissue in Pts 01 and 02 and was in the middle of the range for the other four autopsy patients (**Figure 6B**).

As with the total TCR sequences from the native lung that appear in both native and graft tissue (**Figure 3C**), those COVID-associated (**Supplemental Figure 3C**) and GLIPH2-defined putative SARS-CoV-2-specific (**Supplemental Figure 4C**) sequences appearing in both native and graft tissue also showed a linear inverse correlation between their cumulative frequency in native lung and time from disease presentation to death, although with low R^2^ values (0.45-0.46). Moreover, linear regression analysis was performed correlating the cumulative frequency of total TCR clones with the cumulative frequency of COVID-associated TCRs (**Supplemental Figure 5A**), GLIPH2-defined TCRs (**Supplemental Figure 5B**), or combined COVID-relevant TCRs (**Supplemental Figure 5C**) that are identified in the native lung and shared between native and graft tissue. While these data must be interpreted cautiously due to the lower R^2^ values (0.27-0.48), the result is in line with the hypothesis that a more systemic T cell response to the SARS-CoV-2 virus is associated with faster disease course.

### SARS-CoV-2-specific TCR sequences in transplant patients lack a donor HLA-restricted pattern, regardless of distribution in allograft or native tissues

**Figure 7:**
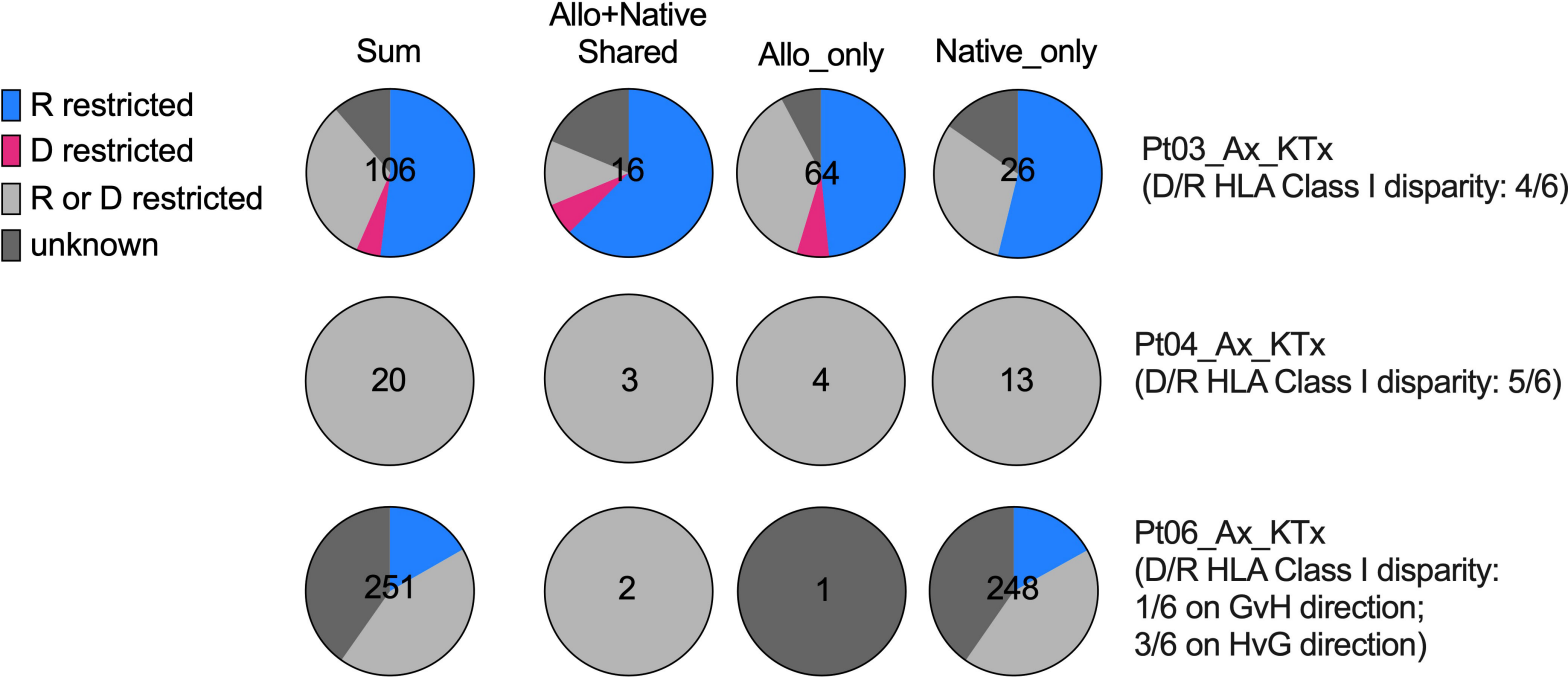
HLA restriction analysis of SARS-CoV-2-specific TCRs defined by bio_identity (CDR3 + v + j amino acid) overlap with MIRA database in Pts03, 04 and 06, from whom donor (“D”) and recipient (“R”) HLA typing information is available. Proportions of SARS-CoV-2-specific TCRs characterized as “R restricted”, “D restricted”, “R or D restricted” and “unknown” are shown in the pie charts for each patient per row, with the first column showing all SARS-CoV-2-specific TCRs and the second, third and fourth columns showing SARS-CoV-2-specific TCRs detectable in both allograft and native tissues, in allograft only and in native tissues only, respectively. The characterization of SARS-CoV-2-specific TCRs in the “R restricted”, “D restricted”, “R or D restricted” and “unknown” categories is described in **Table 3**.

HLA restriction is the cornerstone of human T cell recognition of antigen peptide (31). Transplantation adds more complexity given the coexistence of donor graft cells and recipient cells that have antigen-presenting capacity. In order to understand the potential effect of HLA restriction on SARS-CoV-2-specific TCR repertoire formation after transplantation, we first categorized SARS-CoV-2-specific TCR sequences in our cohort defined by bio_identity (CDR3 + V + J amino acid) overlap with MIRA database into 3 groups: 1) present in both allograft and at least one native tissue; 2) present in allograft only; 3) present in native tissue(s) only. This analysis was performed in Pts 03, 04 and 06, for whom both donor and recipient HLA typing information is available. The MIRA dataset is largely skewed towards MHC Class I restricted TCRs (approximately 154,000) compared to MHC Class II restricted TCRs (approximately 6,800) (21). In fact, all SARS-CoV-2-specific TCRs we identified in Pts 03, 04, and 06 overlapped with the MHC Class I restricted collection of MIRA dataset, so we focused on HLA-A, B, and C alleles in our subsequent analysis (**Figure 7**, **Table 3** and **Supplemental Table 1**).

**Table 3:** HLA restriction analysis of SARS-CoV2-specific TCRs identified by immuneCODE MIRA database in Pt03 as a representative example. Incomplete list of TCRs. For complete list see **Supplemental Table 1**.

Pt03_Ax_KTx	D vs R HLA Class I disparity: 4/6	Recipient HLA		A2	A3	B61	B65	C8	C10		
		Donor HLA		A33	AX	B53	B65	C8	C4		
sample_name	**bio_identity** (those identified in at least two subjects of MIRA dataset were highlighted in green)	Subject	Age	HLA.A	HLA.A.1	HLA.B	HLA.B.1	HLA.C	HLA.C.1	**Putative categories for HLA restriction**	**Putative restricted alleles**
shared_Allo_Nat	CASSQGNEQFF+TCRBV03-01/03-02+TCRBJ02-01	9541	28	A*02:01	A*03:01	B*07:02	B*08:01	C*07:01	C*07:02	R restricted	
shared_Allo_Nat	CAISESSYEQYF+TCRBV10-03+TCRBJ02-07	19617	21	A*01:01	A*02:01	B*35:02	B*39:01	C*04:01	C*07:02	R or D restricted	
shared_Allo_Nat	CASSIGGETQYF+TCRBV19-01+TCRBJ02-05	20655	41	A*11:01	A*68:01	B*35:01	B*35:03	C*03:03	C*04:01	D restricted	
shared_Allo_Nat	CATSRQGGTDTQYF+TCRBV15-01+TCRBJ02-03	1565927	65	A*03:01:01	A*31:01:02	B*45:01:01	B*57:03:01	C*07:18:01	C*16:01:01	R restricted	A*03:01
shared_Allo_Nat	CATSRQGGTDTQYF+TCRBV15-01+TCRBJ02-03	273	49	A*02:01:01	A*03:01:01	B*07:02:01	B*18:01:01	C*07:01:01	C*07:02:01		
shared_Allo_Nat	CATSRQGGTDTQYF+TCRBV15-01+TCRBJ02-03	2845	N/A	A*03:01:01	A*24:02:01	B*07:02:01	B*57:01:01	C*06:02:01	C*07:02:01		
shared_Allo_Nat	CATSRQGGTDTQYF+TCRBV15-01+TCRBJ02-03	535	28	A*03:01:01	A*24:02:01	B*08:01:01	B*14:02:01	C*03:04:01	C*08:02:01		
shared_Allo_Nat	CATSRQGGTDTQYF+TCRBV15-01+TCRBJ02-03	19830	24	A*02:01	A*03:01	B*27:05	B*40:01	C*03:04	C*07:04		
shared_Allo_Nat	CATSRQGGTDTQYF+TCRBV15-01+TCRBJ02-03	1684	72	A*03:01:01	A*03:01:01	B*15:01:01	B*35:03:01	C*03:03:01	C*04:01:01		
shared_Allo_Nat	CATSRQGGTDTQYF+TCRBV15-01+TCRBJ02-03	3821	71	A*02:01:01	A*03:01:01	B*13:02:01	B*14:02:01	C*06:02:01	C*08:02:01		
Allo only	CASSLGGSQPQHF+TCRBV28-01+TCRBJ01-05	14758	30	A*02:01	A*33:03	B*53:01	B*58:01	C*03:02	C*06:02	R or D restricted	
Allo only	CASSLSHTDTQYF+TCRBV27-01+TCRBJ02-03	20795	23	A*02:01	A*02:01	B*40:01	B*57:01	C*03:04	C*06:02	R restricted	
Allo only	CASSRRNEQFF+TCRBV12-03/12-04+TCRBJ02-01	2394	N/A	A*02:03:01	A*11:01:01	B*39:01:01	B*40:01:02	C*07:02:01	C*07:02:01	R restricted	
Allo only	CASSVGETQYF+TCRBV09-01+TCRBJ02-05	14758	30	A*02:01	A*33:03	B*53:01	B*58:01	C*03:02	C*06:02	R restricted	A*02:01
Allo only	CASSVGETQYF+TCRBV09-01+TCRBJ02-05	19830	24	A*02:01	A*03:01	B*27:05	B*40:01	C*03:04	C*07:04		
Allo only	CASSVGETQYF+TCRBV09-01+TCRBJ02-05	19617	21	A*01:01	A*02:01	B*35:02	B*39:01	C*04:01	C*07:02		
Allo only	CASSVGETQYF+TCRBV09-01+TCRBJ02-05	19943	45	A*02:01	A*02:01	B*35:03	B*44:02	C*04:01	C*05:01		
Allo only	CASSVGETQYF+TCRBV09-01+TCRBJ02-05	10943	21	A*02:01	A*26:01	B*44:02	B*52:01	C*03:03	C*05:01		
Allo only	CASSVGETQYF+TCRBV09-01+TCRBJ02-05	10881	39	A*02:01	A*23:17	B*15:03	B*57:02	C*02:10	C*14:03†		
Allo only	CASSVGETQYF+TCRBV09-01+TCRBJ02-05	19384	33	A*02:01	A*24:02	B*15:17	B*40:01	C*04:82	C*07:01		
Allo only	CASSVGETQYF+TCRBV09-01+TCRBJ02-05	19830	24	A*02:01	A*03:01	B*27:05	B*40:01	C*03:04	C*07:04		
Allo only	CASSVGETQYF+TCRBV09-01+TCRBJ02-05	19617	21	A*01:01	A*02:01	B*35:02	B*39:01	C*04:01	C*07:02		
Allo only	CASSVGETQYF+TCRBV09-01+TCRBJ02-05	19830	24	A*02:01	A*03:01	B*27:05	B*40:01	C*03:04	C*07:04		
Allo only	CASSVGETQYF+TCRBV09-01+TCRBJ02-05	19943	45	A*02:01	A*02:01	B*35:03	B*44:02	C*04:01	C*05:01		
Allo only	CASSVGETQYF+TCRBV09-01+TCRBJ02-05	4423	37	A*01:01	A*02:01	B*15:01	B*40:01	C*03:04	C*04:01		
Allo only	CASSVGETQYF+TCRBV09-01+TCRBJ02-05	20795	23	A*02:01	A*02:01	B*40:01	B*57:01	C*03:04	C*06:02		
Allo only	CASSVGETQYF+TCRBV09-01+TCRBJ02-05	19830	24	A*02:01	A*03:01	B*27:05	B*40:01	C*03:04	C*07:04		
Allo only	CASSVGETQYF+TCRBV09-01+TCRBJ02-05	1499	53	A*02:10	A*11:01:01	B*39:01:01	B*40:06:01	C*07:02:01	C*08:01:01		
Allo only	CASSVGETQYF+TCRBV09-01+TCRBJ02-05	20300	28	A*02:01	A*29:02	B*07:02	B*44:03	C*07:02	C*16:01		
Allo only	CASNGGYSNQPQHF+TCRBV06-05+TCRBJ01-05	7717	51	A*01:01:01	A*11:01:01	B*07:02:01	B*08:01:01	C*07:01:01	C*07:02:01	unknown	
Native only	CASSLGSGANVLTF+TCRBV12-X+TCRBJ02-06	273	49	A*02:01:01	A*03:01:01	B*07:02:01	B*18:01:01	C*07:01:01	C*07:02:01	R restricted	
Native only	CASSQDGATNEKLFF+TCRBV04-03+TCRBJ01-04	19617	21	A*01:01	A*02:01	B*35:02	B*39:01	C*04:01	C*07:02	R or D restricted	
Native only	CASSYSSNTEAFF+TCRBV06-05+TCRBJ01-01	19617	21	A*01:01	A*02:01	B*35:02	B*39:01	C*04:01	C*07:02	R or D restricted	
Native only	CASSLSTDTQYF+TCRBV05-04+TCRBJ02-03	19617	21	A*01:01	A*02:01	B*35:02	B*39:01	C*04:01	C*07:02	R restricted	A*02:01
Native only	CASSLSTDTQYF+TCRBV05-04+TCRBJ02-03	20300	28	A*02:01	A*29:02	B*07:02	B*44:03	C*07:02	C*16:01		
Native only	CASSLSTDTQYF+TCRBV05-04+TCRBJ02-03	4423	37	A*01:01	A*02:01	B*15:01	B*40:01	C*03:04	C*04:01		

HLA restriction analysis of SARS-CoV2-specific TCRs identified by immuneCODE MIRA database in Pt03 as a representative example. Incomplete list of SARS-CoV2-specific TCRs defined by sequence overlap with MIRA database subgrouped by their presence in both allograft and native tissues (“shared_Allo_Nat”), only in allograft (“Allo_only”) and only in native tissues (“Native only”) is shown in the first column. Complete list see Supplemental Table 1. The level of donor (“D”) and recipient (“R”) HLA class I disparity is 4/6 for Pt03. Overlapping HLA class I alleles between Pt03 (“D” and “R”) and MIRA subjects were annotated with donor HLA (pink), recipient HLA (blue), or donor/recipient shared HLA (darker grey). One unique TCR sequence can appear in multiple MIRA subjects, several representatives of which are highlighted in green in the second column of this table. These are “public” SARS-CoV-2-specific TCR sequences broadly identifiable in humans, some of which showed a restriction on certain HLA alleles, such as A*03:01 and A*02:01. Each sequence associated with at least one unique MIRA subject will be defined by one of the putative categories: 1) “D restricted” if only annotated with donor HLA and the allele appears in all MIRA subjects with that unique TCR sequence; 2) “R restricted” if only annotated with recipient HLA and the allele appears in all MIRA subjects with that unique TCR sequence; 3) “R or D restricted” if annotated with both donor HLA and recipient HLA, with or without donor/recipient shared HLA alleles; 4) “unknow” restriction if with different HLA-A, B, C from both donor and recipient.

The level of donor and recipient HLA Class I disparity is 4/6 for Pt03, 5/6 for Pt04, and 1/6 in the graft-vs-host (GvH) direction and 3/6 in the host-vs-graft (HvG) direction for Pt06 (**Figure 7**). Since HLA typing of a vast majority of subjects in MIRA database is available, we next screened each HLA allele for overlapping TCRs between our patients and MIRA subjects and annotated them with donor HLA, recipient HLA, or donor/recipient shared HLA (**Table 3** and **Supplemental Table 1**). We frequently found that one unique sequence appeared in multiple MIRA subjects, several representatives of which are highlighted in green in the second column of **Table 3**. These are “public” SARS-CoV-2-specific TCR sequences broadly identifiable in humans, some of which showed a clear restriction on certain HLA alleles, such as A*02:01, A*03:01, A29*02, B*35:02, and C*04:01 (**Table 3** and **Supplemental Table 1**). Therefore, each sequence associated with at least one unique MIRA subject will be defined by one of the putative categories below: 1) donor HLA restricted if only annotated with donor HLA and the allele appears in all MIRA subjects with that unique TCR sequence; 2) recipient HLA restricted if only annotated with recipient HLA and the allele appears in all MIRA subjects with that unique TCR sequence; 3) donor or recipient HLA restricted if annotated with both donor HLA and recipient HLA, with or without donor/recipient shared HLA alleles; 4) unknown if with different HLA-A, B, C from both donor and recipient (**Table 3** and **Supplemental Table 1**).

Interestingly, from 0% to only a minimal fraction (<5%) of SARS-CoV-2-specific TCRs showed a “donor HLA restricted” pattern in the 3 patients tested (Pts 03, 04, and 06), indicating that direct presentation of viral antigens by donor antigen-presenting cells (APCs) or other donor graft cells and semi-direct presentation of viral antigens by recipient APCs cross-dressing donor HLA are less likely to play a major role in driving host T cell anti-viral response in transplant recipients with severe SARS-CoV-2 infection (**Figure 7**). Instead, a vast majority (60-100%) of SARS-CoV-2-specific TCR sequences in transplant patients, regardless of distribution in allograft or native tissues, present either a “recipient HLA restricted”, or “recipient or donor HLA restricted” pattern, suggesting that indirect presentation of viral antigens by recipient cells can effectively trigger host T cell anti-viral responses in both the host and graft (**Figure 7**).

## Discussion

We herein report the first integrated characterization of histological findings, viral infiltration, and distribution of T cell clones, including those associated with the SARS-CoV-2 virus, in multiple tissues, in recipients of solid organ transplants. Patients with severe COVID-19 often present with organ dysfunction, including dysfunction of grafts in transplant patients with severe disease (14, 15, 32). Given the immunosuppressed state of the recipients as well as the altered immune milieu within the graft itself, it remains unknown whether graft dysfunction in severe disease is related to direct viral injury, systemic inflammation, or rejection. We addressed this question by correlating histological findings and lab values with clinical course and inflammatory markers and by comparing the viral infiltration and distribution of TCR clones between graft and native tissues. While our study specifically examines transplant recipients, it is also the first analysis of its kind in any population.

Only two existing studies, one still in pre-print, have mapped viral RNA throughout various tissues in autopsy tissue from patients who died of COVID-19. These studies showed that, while the highest viral load existed in the lungs as expected, viral RNA was also identified throughout the remainder of the body by PCR, with variable levels found in heart (33, 34). In the report most relevant to our findings, Van Cleemput et al. in a study of 13 patients in Belgium who died of COVID-19, 4 of whom were variably immunosuppressed (but none transplanted), found viral PCR positivity throughout both lungs and extrapulmonary tissue (although variably in extrapulmonary tissue) and higher viral loads in blood and lung of patients who succumbed more quickly. Additionally, viral RNA was sometimes found without the concurrent finding of SARS-CoV-2 virions, and viral mutations were identified throughout various extrapulmonary tissues in patients in whom disease course was prolonged (35). Regarding TCR clones specific for the SARS-CoV-2 virus, previous studies have utilized TCR repertoire profiling in blood to obtain biological insights into COVID-19 infection (36), including enrichment of SARS-CoV-2-specific TCRs in the blood of patients with more severe disease (37). However, local tissue coordination of cellular and humoral immune response against SARS-CoV-2 is critical for developing immunological memory necessary for viral protection, including memory responses, in the periphery (38). It is known that viral-specific clones can be identified in various tissues by TCR sequencing (37, 39, 40) or by other methods, such as T cell epitope mapping (34). However, it is largely unexplored how TCR repertoire distribution in association with viral load throughout the body may help to interpret the anti-viral responses in immunocompromised transplant patients with COVID-19 infection.

Our results are characterized by the following major findings. First, transplant patients with severe COVID-19 disease (characterized by either death or prolonged course with respiratory failure requiring intubation) had the same elevated inflammatory markers (IL-6, CRP, ESR, fibrinogen, D-dimer, ferritin, and LDH) observed in non-transplant patients (26). Graft histology of COVID-positive renal transplant patients with lab evidence of graft injury (elevated creatinine) showed acute kidney injury +/- viral injury in those with severe disease, but those with asymptomatic or mild disease showed rejection, calcineurin inhibitor damage, or thrombotic microangiopathy. In other words, renal graft dysfunction in the setting of severe COVID-related disease was related to severe systemic inflammation +/- direct viral injury, while renal graft dysfunction in those without severe disease was secondary to unrelated processes. Similarly, liver injury in those with severe disease was related to shock. The one liver graft among those with severe disease showed mild rejection, but this patient was recovering from a previously diagnosed rejection episode predating the COVID infection, and there were no other indications of rejection based on differences in repertoire or clonal dominance in the graft versus native tissues (see below). All lung histology showed severe inflammation, whether graft or native, and heart histology showed myocardial fibrosis as a consequence of tissue injury in both graft and native heart, with possible contribution of viral infection and systemic inflammation. These findings are significant because they identify the severe systemic inflammatory response as the most likely etiology of graft injury in these patients.

Second, TCR sequences differ markedly between patients but are broadly shared between native organs and allograft within each patient, suggesting the effect of HLA restriction on shaping TCR repertoire and an overall systemic TCR response to the virus within individual patients with SARS-CoV-2 infection. While greater TCR sharing might be expected within the tissues of an individual patient compared to tissues from another individual, it is notable that there was not a markedly different set of dominant TCR sequences in the allograft compared to the native organs, even for the patients with histological evidence of rejection, suggesting strongly that graft damage in these patient was not related to an alloresponse, which we have found to be associated with predominance of alloreactive T cell clones in rejecting allografts (23, 27).

Third, in association with viral load through the body, analysis of repertoire distribution of COVID-associated TCR clones, identified by clonal overlap with COVID T-MAP™ database and GLIPH2-based cluster formation with MIRA dataset, is also indicative of a systemic response.

Cumulative frequencies of total COVID-associated TCRs and putative SARS-CoV-2-specific TCRs identified by GLIPH2 were comparable in lung, liver, kidney or even the heart, regardless of allogeneic or native derivation. This diffuse detection of virus and COVID-associated TCRs, including in the graft, also emphasizes the systemic nature of the infection in those with severe disease who died and shows that the graft is included in the systemic infection and immune response.

The lack of a clear positive correlation between viral PCR and cumulative frequency of COVID-associated clones is likely reflective of several contributors: 1) the PCR identification of viral RNA inside host APCs within the tissues rather than actual viral infiltration, as has been shown in at least one study (35), 2) better clearance of the virus by the host immune response in the organs that are less severely damaged, and 3) a limitation of the Adaptive Biotechnologies COVID library. It is also possible that observing no significant difference in viral load between organs despite only lungs reaching the highest category of viral load is due to heterogenous clinical sampling. On the other hand, the lack of correlation between viral load and cumulative frequency of COVID-associated clones might instead reflect the true physiology of the immune response, in which higher viral loads in the lung combined with the same (or higher) cumulative frequency of COVID-specific TCRs in extrapulmonary organs (especially heart once GLIPH2 is added) indicates that the systemic T cell response to the virus might actually be protective against viral injury in these tissues. For example, our finding in Pt04 of a high percentage of COVID-associated TCRs by cumulative frequency in native kidney, liver, and lung, but lower viral loads in native kidney and liver may indicate effective local elimination of virus by COVID-associated TCRs in native kidney and liver, but not in native lung. A similar pattern was described in non-transplant patients by Park et al., who found that the T cell exposed motif repertoire found in the hearts of patients infected with the SARS-CoV-2 virus was highly abundant but less diverse than that in other organs, suggesting that there might be a targeted anti-viral response in the heart that was protective (34). However, the association we observed of native organ injury (e.g., heart) with large numbers of SARS-CoV-2-reactive, host HLA-restricted TCRs is consistent with the possibility that this systemic T cell response to the virus is destructive of recipient organs. While donor-recipient HLA disparity may protect the donor graft from direct cytotoxic T cell attack, the allograft may be damaged by local or systemic cytokine release and vascular leak mediated by activated myeloid cells, resulting in the observed pathology in lung and kidney allografts in our study.

Fourth, the significant differences in TCR sequences between patients is consistent with the role that HLA restriction plays in shaping the TCR repertoire. Regarding HLA restriction, there was no significant difference between SARS-CoV-2-specific TCR clones found in the graft versus native tissue in the patients for whom we have this information (Pts 03, 04, and 06). It is striking to find that from 0% to up to 5% of SARS-CoV-2-specific TCRs showed a solely “donor HLA restricted” pattern and that a vast majority present either a “recipient HLA restricted” or a “recipient or donor HLA restricted” pattern, thus potentially explaining protection of the graft from a harmful T cell response to the virus. The fact that some unique SARS-CoV-2-specific sequences identified in one of our transplant patients also appear in multiple MIRA subjects further suggests that they are “public” SARS-CoV-2-specific TCR clones that are broadly identifiable in infected populations, many of which showed a clear HLA Class I allele restriction, such as A*02:01, A*03:01, A29*02, B*35:02, and C*04:01.

Finally, our data suggest that greater sharing of TCR clones throughout a patient’s tissues and, by inference, a stronger anti-viral T cell response, might correlate with the intensity of disease (defined herein as time from symptom onset to death). Since the two patients (Pts03 and 04) with the highest percentage of clones shared between tissues were notable for having the shortest diagnosis to death times among our autopsy cohort (2 and 7 days respectively versus 22, 22, 16 and 33 days for the other patients), and mindful of the finding elsewhere that the SARS-CoV-2 virus developed greater mutations within the host in patients with longer-lasting infections (35), we hypothesized that the greater T cell clone overlap in the patients with shortest disease courses might be related to having fewer viral mutations. This is supported by our JSD data, in which there was no significant difference in the JSD for repertoire similarity between graft and native tissue within five of the six autopsy patients, but Pt03 was an outlier with significantly lower JSD (greater similarity of TCR repertoires).

We are aware of several potential weaknesses in our analysis, including the small number of patients, lack of inflammatory marker measurement in some patients, and lack of donor and recipient HLA typing for all. We also recommend caution when generalizing our findings in immunosuppressed patients for immunocompetent patients and in generalizing our results in patients with severe disease for patients with more mild disease. However, our results stand as the only existing analysis of systemic histology findings, viral load quantification, and TCR repertoire and HLA restriction analysis in both allograft and native tissues throughout the body in multiple types of organ transplant recipients. When these data are considered together with clinical course and serum inflammatory markers, they shed new light on SARS-CoV-2 infection as a systemic event, in which both the virus and the immune response to the virus are identified throughout all tissues assessed, and the severity of disease is related not to direct viral infection but most likely to the systemic inflammatory response. Our data suggest that the immune response might be helpful in controlling the viral infection in extrapulmonary organs while also causing damage. For transplant patients specifically, we find that graft dysfunction in the setting of severe COVID infection is related not to rejection or to greater viral invasion but to the systemic inflammatory response. We also find that the systemic viral infection and the systemic immune response to the virus is comparably observed in allogeneic and native tissues, despite the unique immune milieu in the graft, the differing HLA of the graft tissue, and the potential for an alloresponse within the graft. Finally, this indicates that graft dysfunction in transplant recipients with severe COVID disease appears to be secondary to the severe inflammatory disease rather than direct viral damage or rejection. This finding may be helpful to clinicians managing transplant recipients with severe COVID-19 infections.

## Data availability statement

The raw sequence data for the 35 generated TCR libraries were deposited at the Sequence Read Archive with accession numbers listed below under BioProject PRJNA909270: SRR22549093, SRR22549092, SRR22549081, SRR22549070, SRR22549064, SRR22549063, SRR22549062, SRR22549061, SRR22549060, SRR22549059, SRR22549091, SRR22549090, SRR22549089, SRR22549088, SRR22549087, SRR22549086, SRR22549085, SRR22549084, SRR22549083, SRR22549082, SRR22549080, SRR22549079, SRR22549078, SRR22549077, SRR22549076, SRR22549075, SRR22549074, SRR22549073, SRR22549072, SRR22549071, SRR22549069, SRR22549068, SRR22549067, SRR22549066, SRR22549065. Analysis code used to analyze TCR-β bulk DNA-seq data and calculate total productive template counts, unique sequence counts, clonality, R20, JSD, and perform GLIPH2 clustering and mapping to MIRA dataset, as well as HLA restriction analysis is available at https://github.com/jfccti/COVID-TCR-SOT.git. Reference TCR repertoire data from SARS-CoV-2-infected subjects, and the MIRA dataset (release 002.1) are publicly available from the ImmuneAccess database (https://clients.adaptivebiotech.com/pub/covid-2020).

## Ethics statement

The study was approved by the Columbia University Institutional Review Board (IRB# AAAT1929). All subjects or legal guardians provided their written, informed consent and assent when appropriate.

## Author contributions

DR, BC, SM, and RJ coordinated sample collection and analysis. DR collected and analyzed clinical information. SL and IB contributed histological analysis and images. JF, ZF, WJ, and MS analyzed T cell receptor data. JF, MS, and JW wrote the manuscript. JF and JW conceived and oversaw the entire project. All authors contributed to the article and approved the submitted version.

## Supplementary material

Supplementary Figure 1 Total productive TCR template counts **(A)**, unique sequence counts **(C)**, clonality **(E)**, and R20 **(G)** in different organs subgrouped by allografts (squares) and native tissues (circles) for autopsy (Ax) specimens of Pts01-06. Pt01 liver graft was diagnosed as rejection. Total productive TCR template counts **(B)**, unique sequence counts **(D)**, clonality **(F)**, and R20 **(H)** in kidney or heart biopsies (Bx) of Pts07-14 subgrouped by rejection status. No statistical significance was determined by Two-way ANOVA, followed by Tukey’s multiple comparisons test, when two factors are involved for multiple groups. No statistical significance was determined by Mann-Whitney U test between compiled allograft and native tissues regardless of organ types for Pts01-06 nor between compiled rejection and non-rejection biopsies for Pts07-14.

Supplementary Figure 2 Total productive template counts **(A)** and unique sequence counts **(C)** of COVID-associated TCRs in different organs subgrouped by allografts (squares) and native tissues (circles) for autopsy (Ax) specimens of Pts01-06. Pt01 liver graft was diagnosed as rejection. Total productive template counts **(B)** and unique sequence counts **(D)** of COVID-associated TCRs in kidney or heart biopsies (Bx) of Pts07-14 subgrouped by rejection status. Fraction of unique COVID-associated TCRs among all unique TCRs in different organs of Pts01-06 **(E)** and in biopsies of Pts07-14 **(F)**. No statistical significance was determined by Two-way ANOVA, followed by Tukey’s multiple comparisons test, when two factors are involved for multiple groups. No statistical significance was determined by Mann-Whitney U test between compiled allograft and native tissues regardless of organ types for Pts01-06 nor between compiled rejection and non-rejection biopsies for Pts07-14.

Supplementary Figure 3
**(A)** Quantification of the number of unique COVID-associated TCR sequences for each patient (shown in the center of each pie chart) present in the allograft (“allo”) only, native tissue (“native”) only, the allograft plus some native tissues, and the allograft plus all native tissues. **(B)** Quantification of the cumulative frequency of COVID-associated TCR sequences within the allograft (left panel) or native lung (right panel) of each patient subgrouped by their presence in “allo only”, “native only”, “allo + some native” and “allo + all native”. **(C)** Association between cumulative frequency of COVID-associated TCRs detected in both allograft and native tissues within native lung and days from COVID-19 diagnosis to death is shown. Linear regression R^2^ = 0.4635.

Supplementary Figure 4
**(A)** Quantification of the number of unique GLIPH2-defined putative SARS-CoV-2-specific TCRs for each patient (shown in the center of each pie chart) present in the allograft (“allo”) only, native tissue (“native”) only, the allograft plus some native tissues, and the allograft plus all native tissues. **(B)** Quantification of the cumulative frequency of GLIPH2-defined putative SARS-CoV-2-specific TCRs within the allograft (left panel) or native lung (right panel) of each patient subgrouped by their presence in “allo only”, “native only”, “allo + some native” and “allo + all native”. **(C)** Association between cumulative frequency of GLIPH2-defined putative SARS-CoV-2-specific TCRs appeared in both allograft and native tissues in native lung and days from COVID-19 diagnosis to death is shown. Linear regression R^2^ = 0.4525.

Supplementary Figure 5
**(A)** Association of cumulative frequencies of COVID-associated TCRs vs total TCRs appeared in both allograft and native tissues within native lung is shown. Linear regression R^2^ = 0.27. **(B)** Association of cumulative frequencies of GLIPH2-defined putative SARS-CoV-2-specific TCRs vs total TCRs appeared in both allograft and native tissues within native lung is shown. Linear regression R^2^ = 0.3588. **(C)** Association of cumulative frequencies of COVID-associated and GLIPH2-defined putative SARS-CoV-2-specific TCRs vs total TCRs appeared in both allograft and native tissues within native lung is shown. Linear regression R^2^ = 0.4839.
