# Supplementary figures and images for: T cell repertoire profiling in allografts and native tissues in recipients with COVID–19 after solid organ transplantation: Insight into T cell–mediated allograft protection from viral infection
Source: Front Immunol. 2022 Dec 14;13:1056703. doi: 10.3389/fimmu.2022.1056703 (PMC9795050; doi:10.3389/fimmu.2022.1056703)

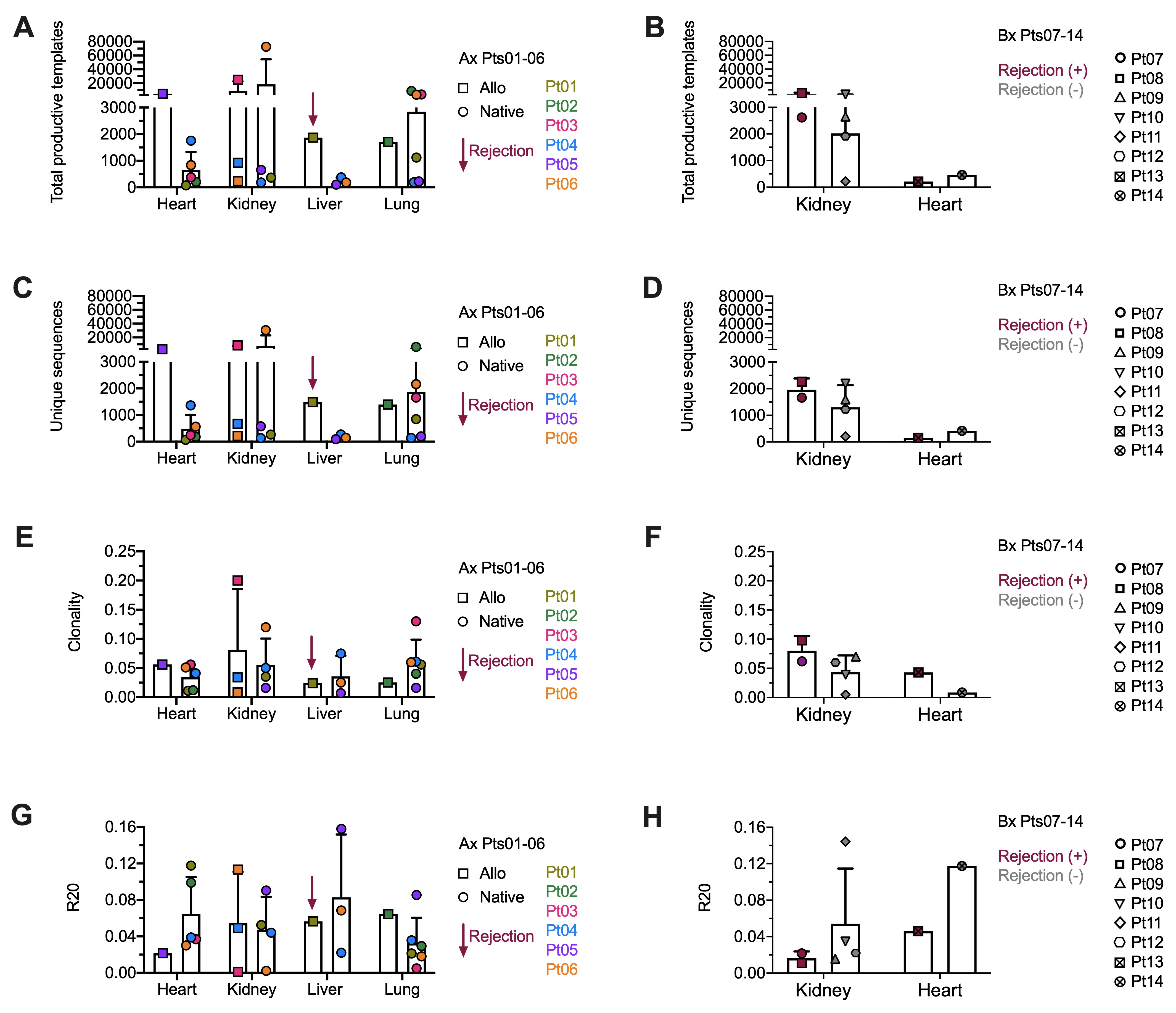

Supplement: Supplementary Figure 1 — Total productive TCR template counts (A), unique sequence counts (C), clonality (E), and R20 (G) in different organs subgrouped by allografts (squares) and native tissues (circles) for autopsy (Ax) specimens of Pts01-06. Pt01 liver graft was diagnosed as rejection. Total productive TCR template counts (B), unique sequence counts (D), clonality (F), and R20 (H) in kidney or heart biopsies (Bx) of Pts07-14 subgrouped by rejection status. No statistical significance was determined by Two-way ANOVA, followed by Tukey’s multiple comparisons test, when two factors are involved for multiple groups. No statistical significance was determined by Mann-Whitney U test between compiled allograft and native tissues regardless of organ types for Pts01-06 nor between compiled rejection and non-rejection biopsies for Pts07-14. [file Image_1.jpg]

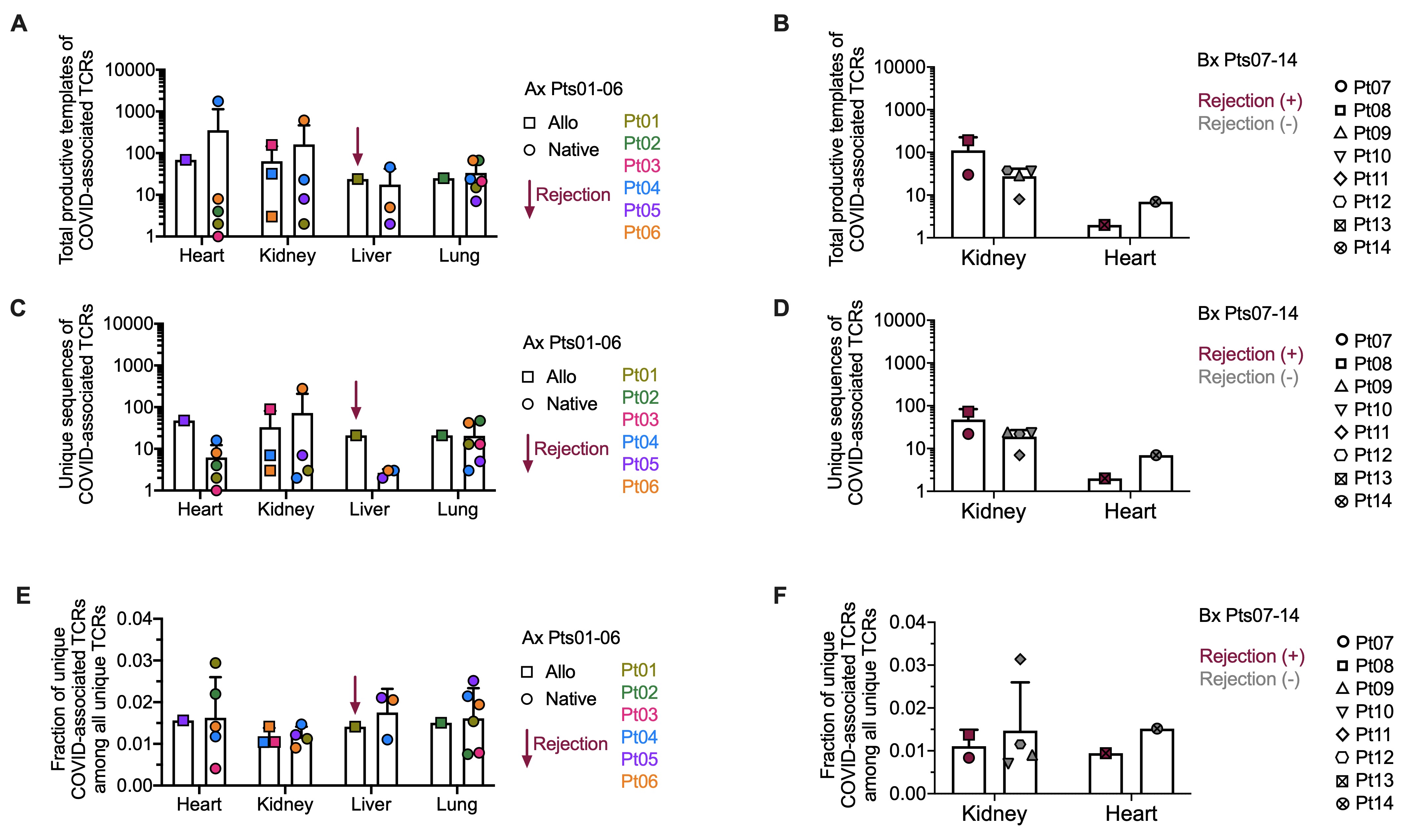

Supplement: Supplementary Figure 2 — Total productive template counts (A) and unique sequence counts (C) of COVID-associated TCRs in different organs subgrouped by allografts (squares) and native tissues (circles) for autopsy (Ax) specimens of Pts01-06. Pt01 liver graft was diagnosed as rejection. Total productive template counts (B) and unique sequence counts (D) of COVID-associated TCRs in kidney or heart biopsies (Bx) of Pts07-14 subgrouped by rejection status. Fraction of unique COVID-associated TCRs among all unique TCRs in different organs of Pts01-06 (E) and in biopsies of Pts07-14 (F). No statistical significance was determined by Two-way ANOVA, followed by Tukey’s multiple comparisons test, when two factors are involved for multiple groups. No statistical significance was determined by Mann-Whitney U test between compiled allograft and native tissues regardless of organ types for Pts01-06 nor between compiled rejection and non-rejection biopsies for Pts07-14. [file Image_2.jpg]

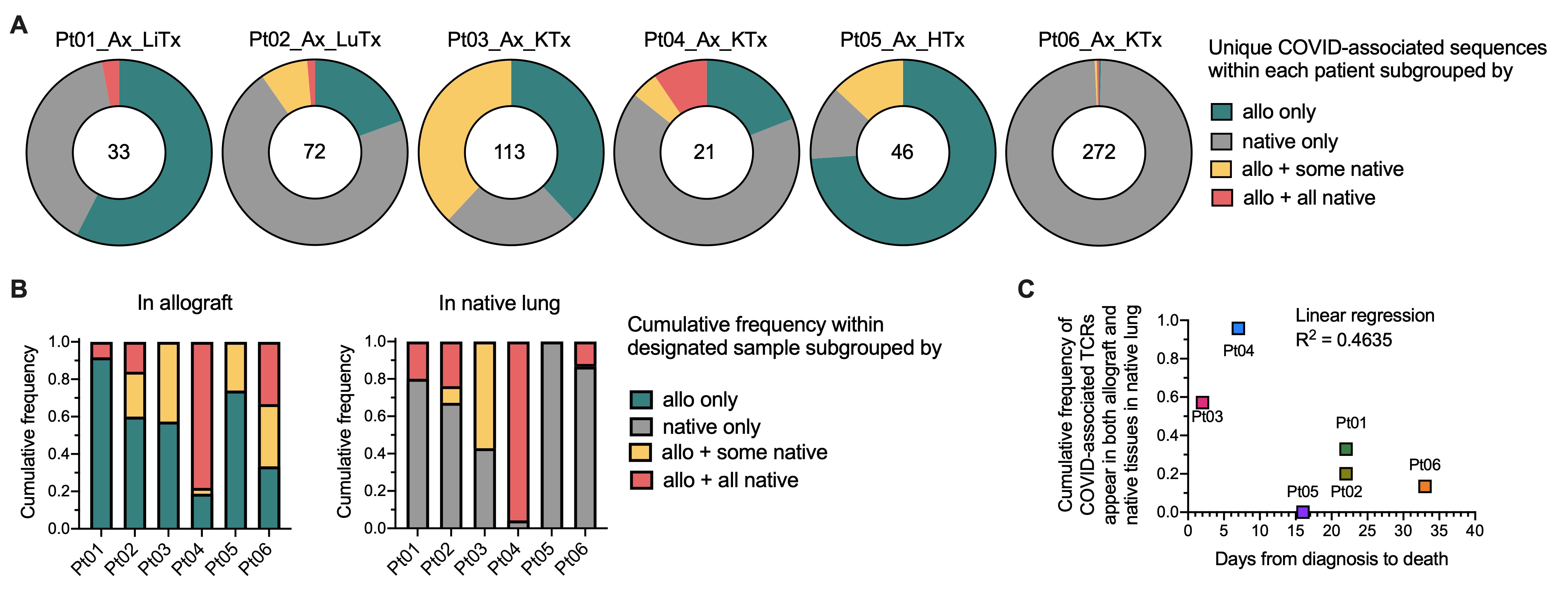

Supplement: Supplementary Figure 3 — (A) Quantification of the number of unique COVID-associated TCR sequences for each patient (shown in the center of each pie chart) present in the allograft (“allo”) only, native tissue (“native”) only, the allograft plus some native tissues, and the allograft plus all native tissues. (B) Quantification of the cumulative frequency of COVID-associated TCR sequences within the allograft (left panel) or native lung (right panel) of each patient subgrouped by their presence in “allo only”, “native only”, “allo + some native” and “allo + all native”. (C) Association between cumulative frequency of COVID-associated TCRs detected in both allograft and native tissues within native lung and days from COVID-19 diagnosis to death is shown. Linear regression R2 = 0.4635. [file Image_3.jpg]

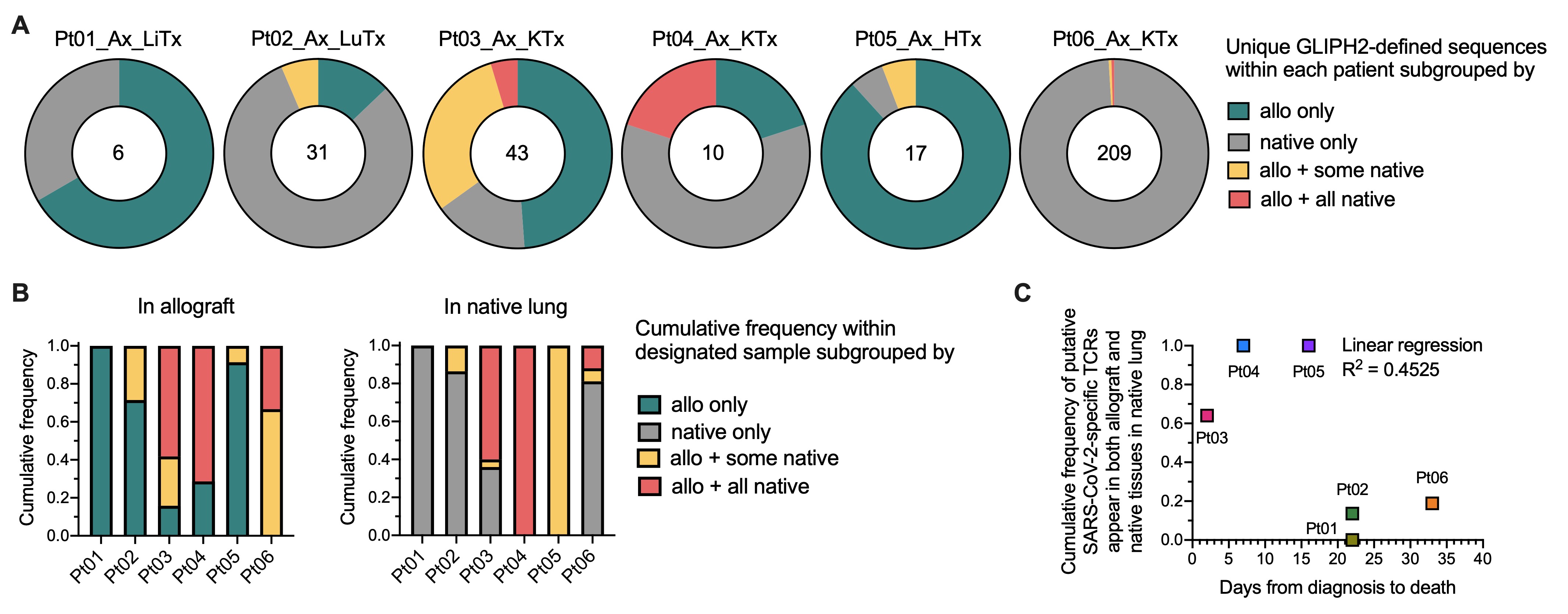

Supplement: Supplementary Figure 4 — (A) Quantification of the number of unique GLIPH2-defined putative SARS-CoV-2-specific TCRs for each patient (shown in the center of each pie chart) present in the allograft (“allo”) only, native tissue (“native”) only, the allograft plus some native tissues, and the allograft plus all native tissues. (B) Quantification of the cumulative frequency of GLIPH2-defined putative SARS-CoV-2-specific TCRs within the allograft (left panel) or native lung (right panel) of each patient subgrouped by their presence in “allo only”, “native only”, “allo + some native” and “allo + all native”. (C) Association between cumulative frequency of GLIPH2-defined putative SARS-CoV-2-specific TCRs appeared in both allograft and native tissues in native lung and days from COVID-19 diagnosis to death is shown. Linear regression R2 = 0.4525. [file Image_4.jpg]

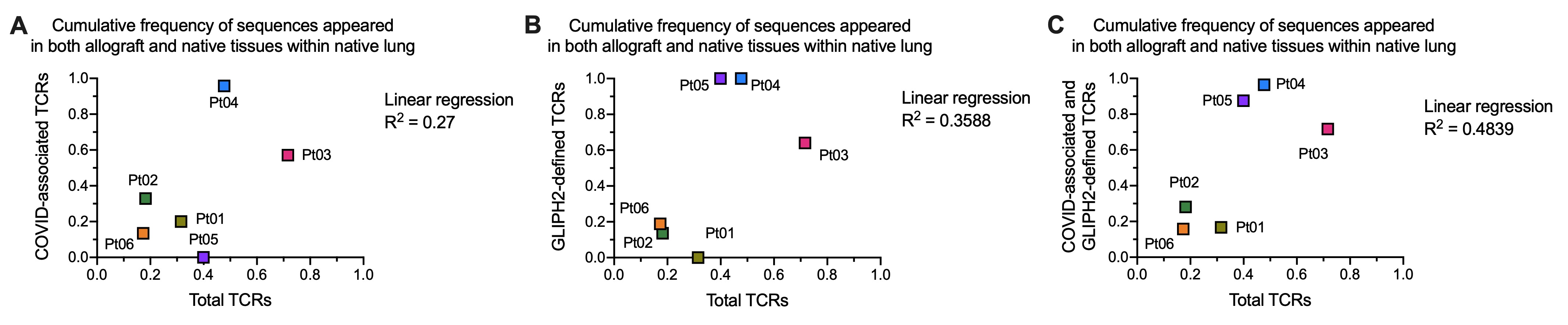

Supplement: Supplementary Figure 5 — (A) Association of cumulative frequencies of COVID-associated TCRs vs total TCRs appeared in both allograft and native tissues within native lung is shown. Linear regression R2 = 0.27. (B) Association of cumulative frequencies of GLIPH2-defined putative SARS-CoV-2-specific TCRs vs total TCRs appeared in both allograft and native tissues within native lung is shown. Linear regression R2 = 0.3588. (C) Association of cumulative frequencies of COVID-associated and GLIPH2-defined putative SARS-CoV-2-specific TCRs vs total TCRs appeared in both allograft and native tissues within native lung is shown. Linear regression R2 = 0.4839. [file Image_5.jpg]
